# On the Functional Overlap between Complement and Anti-Microbial Peptides

**DOI:** 10.3389/fimmu.2014.00689

**Published:** 2015-01-19

**Authors:** Jana Zimmer, James Hobkirk, Fatima Mohamed, Michael J. Browning, Cordula M. Stover

**Affiliations:** ^1^Department of Infectious Diseases – Medical Microbiology and Hygiene, Ruprecht-Karls-University of Heidelberg, Heidelberg, Germany; ^2^Department of Academic Endocrinology, Diabetes and Metabolism, Hull York Medical School, University of Hull, Hull, UK; ^3^Department of Infection, Immunity and Inflammation, University of Leicester, Leicester, UK; ^4^Department of Immunology, Leicester Royal Infirmary, Leicester, UK

**Keywords:** immune cells, innate immunity, histidine tag, deficiencies, acute inflammation

## Abstract

Intriguingly, activated complement and anti-microbial peptides share certain functionalities; lytic, phagocytic, and chemo-attractant activities and each may, in addition, exert cell instructive roles. Each has been shown to have distinct LPS detoxifying activity and may play a role in the development of endotoxin tolerance. In search of the origin of complement, a functional homolog of complement C3 involved in opsonization has been identified in horseshoe crabs. Horseshoe crabs possess anti-microbial peptides able to bind to acyl chains or phosphate groups/saccharides of endotoxin, LPS. Complement activity as a whole is detectable in marine invertebrates. These are also a source of anti-microbial peptides with potential pharmaceutical applicability. Investigating the locality for the production of complement pathway proteins and their role in modulating cellular immune responses are emerging fields. The significance of local synthesis of complement components is becoming clearer from *in vivo* studies of parenchymatous disease involving specifically generated, complement-deficient mouse lines. Complement C3 is a central component of complement activation. Its provision by cells of the myeloid lineage varies. Their effector functions in turn are increased in the presence of anti-microbial peptides. This may point to a potentiating range of activities, which should serve the maintenance of health but may also cause disease. Because of the therapeutic implications, this review will consider closely studies dealing with complement activation and anti-microbial peptide activity in acute inflammation (e.g., dialysis-related peritonitis, appendicitis, and ischemia).

## Background

The host defense against microorganisms relies on both innate and adaptive elements. Innate immunity is the first line of defense against a microbial pathogen, which exposes a pathogen-associated molecular pattern or more simply a prokaryotic surface membrane, differing from eukaryotic biphospholipid layers in the complete absence of cholesterol. For an efficient and directed response, complement uses both pattern recognition and missing self-recognition strategies [reviewed by Ref. (1)]. Besides, it involves a highly controlled, rapid cascade, and crosstalks with other biological systems, for example, with Toll-like receptors (2). Control of the complement system is maintained by a group of membrane-anchored proteins and soluble, circulating proteins referred to as complement regulatory proteins. Regulatory proteins can act at different points in the complement cascade and help control complement attack and adjust its severity, propagation, and endpoints to the cellular target (3). Cells expose membrane-anchored proteins like membrane cofactor protein (MCP or CD46), decay accelerating factor (DAF or CD55), complement receptor 1 (CR1 or CD35), and CD59 as complement regulatory proteins (4), while properdin and factor H may become membrane associated and then are thought to fine tune locally the extent of complement activation (5).

Defensins are able to kill or eliminate bacteria, fungi, protozoans, and viruses. α- and β-defensins are synthesized as precursors that are proteolytically cleaved into their anti-microbially active forms (6). Human neutrophil peptides (HNP)1 to HNP3, for example, are found in high concentrations in granules of neutrophils (7) and released by degranulation in response to pro-inflammatory or bacterial stimuli (8). Human defensin (HD)5 and HD6 are present in Paneth cells in the crypts of the small intestine (9), whereas β-defensins are induced in epithelial cells by wounding, bacterial products, or pro-inflammatory cytokines (10–13). Based on the chemotactic effect exerted by anti-microbial peptides, much work was spent on identifying a receptor for their actions. It has now emerged that CCR2 and CCR6 are receptors for β-defensins (14), and that the interaction of, e.g., HD6 with glycosaminoglycans may modulate binding of one or the other to CCR2 (15). There are different ways of LL-37 uptake into a cell. The receptors FPRL-1 and P2X7 are important for LL-37 activity and lead to chemoattraction and Il-1β processing, respectively (16, 17). In contrast, cellular uptake of LL-37 into epithelial cells has been shown to be mediated by atypical endocytic processes (18).

## Complement and Anti-Microbial Peptides Shape the Local Environment

Local production of complement components and their role in the inflammatory microenvironment is a currently emerging field. Most of the complement pathway proteins are synthesized in the liver (19); however, extrahepatic biosynthesis additionally occurs in a variety of other tissues and organs (20). Locally produced complement proteins, finely tuned according to the demands of the local environment, may allow differential regulation of inflammation and cellular activation within these tissues. Complement factor H, besides its hepatic expression, is further expressed at low levels in lung, heart, spleen, brain, eye, kidney, pancreas, placenta, as well as neurons and glial cells (21). Production of complement proteins and their regulators directly at sites of inflammation offers an underestimated variety of functions for complement proteins. So far, several cell types have been found to produce complement proteins including macrophages (22), fibroblasts (23), endothelial cells (24), as well as organ specific cells (25–28). Intriguingly, even cells such as peripheral monocytes that were thought to be incapable of synthesizing complement proteins unless activated have recently been shown to produce C1q (29). This suggests a new role for locally synthesized C1q in the immediate local response to pathogen- or danger-associated molecular patterns (PAMPs or DAMPs). Properdin, the positive regulator of the alternative pathway, is produced by a variety of cell types like neutrophils (30), peripheral blood monocytes (31), endothelial cells (32), and T cells (33). Properdin released by phagocytes was shown to bind to apoptotic and necrotic cells (33, 34), contributing to their direct removal or properdin-mediated complement activation. Likewise, local release of properdin may opsonize and kill microorganisms using the same mechanism, if indeed it can operate as a pattern-recognition molecule in its own right (35).

The role of complement in modulating inflammation and maintaining homeostasis is only recently becoming apparent. Local immune responses can be altered by C5a via modulating the local cytokine milieu, especially via the cytokines IL-17 and IL-23. While C5a has been shown to enhance IL-17F, it limits IL-17 and IL-23 production by macrophages or DCs (36). In agreement with these findings, another report determined that IL-17 levels in experimental asthma are reduced by signaling through C5aR (37). So far, little is known about local synthesis and specific function of complement proteins where produced away from the humoral environment that has led to the well-known diagrams of sequential assemblies and enzymatic cleavages. Due to functional studies, there is increasing evidence that locally produced complement proteins are biologically active and have a significant role in local environment. Local synthesis of complement proteins not only contributes to the systemic pool of complement (38) but also influences local tissue injury and provides a link with the antigen-specific immune response (39). The diverse range of extrahepatic sites for synthesis of complement proteins and their regulators suggests the importance and need for local availability of the proteins. It has been suggested recently that plasma-borne complement activation vs. cellular production of complement components sufficient to form convertases may pursue distinct, compartment-selective, biological functions (40). Understanding the relative importance of local and systemic complement production could help to explain the differential involvement of complement in organ-specific pathology.

Locality of production plays an important role not only for complement proteins but also for anti-microbial peptides. Paneth cells in the small intestine have been shown to release granules into the lumen of the crypts thereby contributing to mucosal immunity (41). Those granules contain proteins that are associated with roles in host defense, including lysozyme (42), secretory phospholipase A2 (43), and α-defensins termed cryptidins (44). Anti-microbial peptides secreted by Paneth cells are important for innate immunity as they protect mitotically active crypt cells from colonization by potential pathogens and confer protection from enteric infection (45). Moreover, secretion into the crypt lumen defines the apical environment of neighboring cells (46).

## Lytic Activities of Complement and Anti-Microbial Peptides

Both complement proteins and anti-microbial peptides share lytic activities. Anti-microbial peptides attack bacteria, fungi, protozoa, and certain viruses by inserting into their membrane manifold causing pore formation and subsequent lysis (47, 48). Due to the cationic character of microbial peptides, electrostatic attraction to the negatively charged phospholipids of microbial membranes occurs resulting in integration into the microbial cell membrane and membrane disruption.

In the absence of regulators, complement proteins contribute to lysis of cells by forming a membrane attack complex (MAC). After cleavage of C5 into C5a and C5b by the highly specific C5 convertase, C5b initiates the terminal complement pathway involving a non-enzymatic assembly of C6, C7, C8, and C9 to form the MAC to cause lysis. Fusion of those proteins brings forth hydrophobic sites that can insert into the membrane to form a transmembrane channel (49). While only one mode of insertion to form a transmembrane channel for the MAC has been described (50), several models exist to explain the insertion of conformationally changed anti-microbial peptides into and across target membranes (51). Pathogens actively interfere with either of these lytic effector processes (52, 53).

Peptides synthesized form the C-terminal portion of complement C3a have inhibitory effect on the growth of *P. aeruginosa, E. coli, B. subtilis*, and *C. albicans*, which does not exceed the activity of equal molar amounts of LL-37 (54). Native human C3a, however, showed inhibitory effect on *C. albicans* growth, which exceeded that of LL-37 at equimolar amounts [50μM; (55)]. While 6 μM C-terminal C3a peptide was needed to observe membrane disruption of *P. aeruginosa* (54), 1 μM native C3a produced leakage of liposomes (55).

## Phagocytic and Chemo-Attractant Activities of Complement and Anti-Microbial Peptides

The peritoneal cavity is a site in which complement and anti-microbial peptides are key components of the innate immune response and have been investigated with regard to peritoneal dialysis (56, 57). Both mesothelium and leukocytes are the source for this production (58). While the opsonophagocytic activity of complement is well known (via C3b/iC3b), recent findings show that LL-37 can modulate the expression of receptors, which determine the extent of the phagocytic response of human macrophages *in vitro* (59). Both components of the innate immune response are thereby able to influence the adaptive immune response by altering the phenotype of phagocytic cells to become more mature, i.e., acquire characteristics, which will make them more potent to present antigen in a suitable, germinal center environment. Chemotactic activity of complement *per se* (via generation of C3a, C5a, and engagement with their receptors, C3aR, C5aR, C5L2) has been described (60). In addition, however, bradykinin, which may be released after activation of kininogen by the lectin pathway of complement activation (61), has chemotactic activity (62). Contact and complement system cooperate in a pro-inflammatory way. Interestingly, β-defensins can bind to chemokine receptors, in particular, CCR6 present on dendritic cells and T cells (14) and CCR2 (see above). Complement C3a and CXCL12 cooperate in the chemotaxis of CD34^+^ progenitor cells in bone marrow, but the receptor has not yet been described, though C3aR has been excluded (60).

## Cell Instructive Roles of Complement and Anti-Microbial Peptides

Anti-microbial peptides and complement are constitutively expressed and are upregulated during inflammation. While anti-microbial peptides are commonly known to be synthesized by epithelial cells to partake in the innate host defense (63), the contribution of complement expression in non-lymphoid cells is not well appreciated yet, although the pattern of expression in crypts follows that of anti-microbial peptides (26). Beyond their chemo-attractant ability, complement and anti-microbial peptides may assume immunoadjuvant, i.e., adaptive immunity supportive, properties (63, 64). The type of cellular response is co-determined by the integration of signaling events triggered by mediators. So complement activation products and anti-microbial peptides, which can alter their expression manifold acutely and remain altered chronically, are relevant determinants of this cell activity (65, 66).

Innate lymphoid cells located in the mucosa contribute to the barrier by releasing IL-22, which stimulates the production of anti-microbial peptides (67). IL-22 is also expressed, in the context of TGF-β, by IL-17A and IL-17F expressing CD4^+^Th17 cells. Synergistically, IL-22 and IL-17A lead to significant induction of mRNA expression for hBD2, S100A7–9 by keratinocytes (68). Because, on its own, IL-17A is a potent stimulator of anti-microbial peptide production (68), those studies reporting a deviation in complement activity, which impact on the Th17 cell population (69, 70), have to be viewed with care. It is likely that a greater component within the immune response is significantly determined by the relative amounts of anti-microbial peptides, which escape attention in the complement field. In this sense, it is a matter of discussion whether the phenotype observed in the properdin-deficient mice when infected with *Listeria monocytogenes* could be significantly influenced by a lack of anti-microbial peptides, which would be due to significantly lower Il-17 levels, which, importantly, do not adequately upregulate during infection (71). C5a and an N-terminal peptide of human lactoferrin with anti-microbial activity, by stimulating macrophages or dendritic cells, respectively, are able to enhance production of Th17 cells (72, 73), which act in a pro-inflammatory, Treg opposing, way.

## Role of Complement and Anti-Microbial Peptides in Endotoxin Clearance

Intact complement activation in the humoral system (blood) is needed for efficient endotoxin clearance (74), while it exerts at the same time a modulatory effect on cellular, pro-inflammatory activity (75). Anti-microbial peptides may have LPS-neutralizing effect, which is important for the beneficial outcome from sepsis (76). Avoiding exhaustion of these systems would obviate the detrimental development of endotoxin tolerance in sepsis. In severe sepsis, significantly lower levels of plasma C3 have been reported (77) and a failure of PBLs to induce defensins *ex vivo* in response to endotoxins (78). Low Vitamin D3 levels have been linked to mortality in sepsis (79). Interestingly, Vitamin D3 promotes production of LL-37 and β-Defensin (80) as well as C2 and C3 (81, 82) *in vitro*. The complement receptor C5aR is upregulated in lung, liver, kidney, and heart during the early phases of sepsis. Blocking of C5aR has been correlated to improved survival in murine models of sepsis (83).

## Monocytes and Macrophages are Distinct Producers for C3 and Anti-Microbial Peptides

Monocytes appear to need LPS stimulation to produce C3 (84), whereas macrophages were shown to produce basal levels of C3 even without stimulation (85, 86). As a recurring point, most of the papers suggest that macrophage differentiation has to have taken place before considerable C3 production occurs (85–92). This observation is also supported by Affymetrix array data (http://www.ncbi.nlm.nih.gov/geoprofiles/60640353), showing more C3 mRNA in macrophages compared to monocytes.

Both monocytes and macrophages are also affected by anti-microbial peptides. The honeybee anti-microbial peptide apidaecin, for example, has been shown to bind both to human macrophages and monocytes (93) without inducing cytotoxic effects. However, apidaecin shows a different subcellular localization in the cytoplasm or in endosomal compartments for macrophages or monocytes, respectively. Besides, the effect upon LPS stimulation differs. Antagonizing LPS-stimulatory effects on both macrophages and monocytes at low concentrations, a high concentration of apidaecin stimulated pro-inflammatory and pro-immune functions of macrophages. Not only for complement production but also for anti-microbial peptides, monocyte to macrophage differentiation plays an important role. The peptide hLF1–11 applied on monocytes during GM-CSF-driven differentiation has been shown to modulate differentiation toward a macrophage subset characterized by both pro- and anti-inflammatory cytokine production and increased responsiveness to microbial structures (94, 95).

Macrophages are considered classically activated (M1) when stimulated by IFNγ or LPS and alternatively activated (M2) when stimulated by IL-4 or IL-13 (96). The arising question is therefore, which subpopulation of macrophages produces C3 predominantly. There were some hints pointing toward M1 macrophages like fact that synthesis of C3 in various organs can be directly upregulated by IFNγ during an inflammatory response (97). In addition, IFNγ can induce C3 synthesis directly (98) as well as stabilize C3 mRNA (99). Recent studies using guinea pigs deficient for complement C3 showed an impaired antibody response to T-dependent antigens (100), a response dependent on M1 macrophages as well. Those data reveal that C3 production is a highly regulated process and can be modulated by a variety of cytokines, determining whether a macrophage will differentiate into an M1 or M2 macrophage and therefore produce more or less C3, respectively.

Anti-microbial peptides were shown to modulate inflammatory responses as well. LL-37, for example, dramatically reduced levels of pro-inflammatory cytokines such as TNF-α and NO in M1 and M2 bone marrow-derived macrophages, whereas anti-inflammatory functions remained unaltered (101). The same effect could also be observed for human THP-1 cells (102). Another example is the Vitamin D inducible LL-37 anti-microbial peptide, which is expressed mainly by M1 macrophages (103). A recent review sheds light on the feature of monocytes and macrophages to respond differently: they are of heterogeneous origin and do not necessarily follow the differentiation pathway of monocyte–macrophage (104).

## Deficiencies of Complement and Anti-Microbial Peptides

In humans, genetic deficiencies of the great majority of complement components have been described, giving insights into their functions in both infectious and non-infectious diseases. It is beyond the scope of this article to give a detailed review of genetically determined deficiencies of the complement system. [For a more comprehensive review, see in Ref. (105) or (106).] Deficiencies of most complement components give rise to increased susceptibility to specific pathogens or groups of pathogens. In broad terms, deficiencies of components of the classical pathway (C1q,r,s, C4, and C2) are associated with infections with encapsulated bacteria, such as *S. pneumoniae* and *N. meningitidis*. Deficiencies of lectin pathway components (MBL, MASP-2, and ficolin) have been associated with increased frequencies of (usually less severe) respiratory infections. However, asymptomatic lectin pathway-deficient individuals have also been described. C3-deficient patients suffer from a broader range of pyogenic infections, including more severe respiratory infections and meningitis (e.g., *S. pneumoniae*, *N. meningitidis, S. pyogenes, H. influenzae, S. aureus)*. Deficiencies of the regulatory proteins properdin and Factor D, as well as of the terminal components of complement activation (C5–C9), are associated with an increase in susceptibility to *Neisserial* infections, reflecting the important role of cytolytic complement activity in the innate immune response against *Neisseriae*. Deficiencies of Factors H and I are associated with increased pyogenic infections (*N. meningitidis, H. influenzae*, and *S. pneumoniae)*. For some complement deficiencies, the lack of complement function in antibacterial immunity may be compensated for by the production of high levels of pathogen-specific IgG antibodies (107). Consequently, the infections may be more prevalent in childhood. Interestingly, deficiencies of some complement components are also associated with non-infectious conditions. For example, deficiencies of C1q,r,s, C4, and C2 are associated with systemic lupus erythematosus (SLE)-like disease, reflecting the important role of the classical complement pathway in clearance of immune complexes from the body. In these complement deficiencies, the autoimmune manifestations may be of greater clinical significance than the increased susceptibility to infections. Similarly, deficiencies of factors H or I most commonly present with atypical hemolytic uremic syndrome. The most obvious example of a non-infectious condition associated with a complement component deficiency is the association between C1 inhibitor deficiency and hereditary angioedema, in which patients suffer from (potentially life threatening) episodic attacks of tissue edema, due to loss of the inhibitory role of C1 inhibitor in cleavage of high molecular weight kininogen to produce bradykinin.

Deficiencies of anti-microbial peptides are less well defined. Anti-microbial peptides play an important role in immune defense in *Drosophila* (108). LL-37-knockout mice have been generated, and are described as having an increased susceptibility to a number of Gram-negative bacterial infections (109–113), suggesting a broad role for anti-microbial peptides in the immune response to infections in mammals. To date, genetic deficiencies of anti-microbial peptides have not been defined in humans. However, reduced expression of anti-microbial peptides in patients has been associated with increased susceptibility to infections of skin and periodontal gingiva (114–116). As we move toward an era in which exome sequencing becomes a feasible approach for defining genetic defects predisposing to immune deficiencies in patients, the significance of deficiencies of anti-microbial peptides in defense against infections may become apparent.

## Role of Complement and Anti-Microbial Peptides in Acute Inflammation

Activation of complement reveals beneficial functions such as pathogen sensing and defense and clearing injured cells on the one hand; however, complement has been shown to play a major role in pathogenesis of various inflammatory processes on the other hand. In response to pathogens or tissue damage, complement is highly capable of inducing all classical signs of inflammation such as redness, pain, hyperthermia, and swelling. Complement products lead to a release of pro-inflammatory mediators, upregulation of adhesion molecules, and increased vascular permeability of endothelial cells (117). Besides the beneficial effect of clearing an infection locally, complement activation may also contribute to a life-threatening systemic inflammatory response (118). Both the classical and alternative complement pathways appear to be activated during sepsis (119) resulting in elevated levels of the complement activation products C3a, C4a, and C5a (120). Among those, C5a appears to be the most harmful molecule (121). Complement activation seems to play a role in acute inflammation in lung and liver, where it has been correlated to acute respiratory distress syndrome and to acute humoral rejection, respectively (122, 123). Part of its detriment complement activation derives from the crosstalk to other activation systems, such as the kininogen pathway and coagulation cascade (124). Besides, systemic complement activation has been confirmed in stroke patients (125). The anaphylatoxins C3a and C5a exert both protective and harmful functions in the central nervous system (126, 127). Direct contact between blood and cerebrospinal fluid in blood–brain barrier dysfunction leads to production of C1q and generation of C3a, and C5a, which in turn contributes to intracranial inflammation by induction of blood–brain barrier damage and increase in vascular permeability (128, 129). Another example for complement activation is ischemia–reperfusion injury. In ischemia and during reperfusion, complement is activated via the classical, the alternative, and the MBL pathway (130–132). Inhibition of the complement cascade greatly reduced myocardial damage after myocardial infarction (133–135). The role of complement in atherosclerosis remains controversial. Several studies revealed a protective role of complement activation in cardiovascular diseases such as atherosclerosis or vasculitis. The protective effect of complement in the pathogenesis of atherosclerosis has been shown by C3^−/−^ mice exhibiting accelerated development of atherosclerosis (136). We have previously reported on the complexity in design and analysis of complement-targeted mouse models (137). However, a recent population based cohort study showed that unlike C3a, C3, and C5a are not associated with atherosclerosis (138). This suggests that C3a and C3 have distinct roles in pathways leading to cardiovascular diseases. In contrast, a murine study reported that systemic inhibition of complement by Crry–CR2 reduced development of atherosclerosis (139).

Anti-microbial peptides play a modulatory role in acute inflammation via modulation of cytokine production, recruitment of immune cells to the site of injury, and enhancement of phagocytosis (140). Stimulation with IL-4 or IL-13 – classical Th2 response cytokines – leads to rapid Paneth cell degranulation and subsequent release of anti-microbial peptides (141). Anti-microbial peptides play an important role in maintaining the skin barrier and protection against infections. This has been experimentally underlined by mice deficient for LL-37 (142). In addition, LL-37, HBD-2, and 3 are highly expressed in epidermal keratinocytes in response to injury or infections of the skin (143). It has been further shown that LL-37 prevents sepsis by directly dampening pro-inflammatory signaling initiated by LPS (102). Therefore, it may also play a role in dialysis-related peritonitis where endotoxins are present. Defects in defensin expression have been shown to contribute to a number of mucosal inflammatory diseases, including necrotizing enterocolitis and inflammatory bowel disease (144). Moreover, differentially regulated expression of epithelial-derived anti-microbial peptides has been shown in acute appendicitis. Arlt et al. (145) showed that the anti-microbial peptide HBD-1 is downregulated in patients with acute appendicitis, whereas HNP1–3, HD5 and HD6, and HBD2 and 3 are upregulated, suggesting that differential regulation of the innate immune system is coincident with altered bacterial diversity.

## The Case of C3a and Other Anti-Microbial Agents

Structural criteria together with functional *in vitro* data suggest that C3a and C4a, but not C5a (all split products of complement activation), may qualify as anti-microbial peptides *per se* (51). C3a (9 kDa), C3a_desarg_, and synthetic peptides derived from C3a were compared to LL-37 (5 kDa when processed) for their inhibitory effect on *E. coli*, *E. faecalis*, and *P. aeruginosa*, their heparin binding, liposome permeabilization and were found to be strikingly similar (146). Structurally, C3a contains α-helical regions characteristic of anti-microbial peptides, which were found represented in proteolytic fragments generated by the enzymatic activities of cells involved in the acute inflammatory response, such as neutrophils and mast cells (147).

Anti-microbial activity and heparin binding ability are described for histidine-rich peptides (148). Histidine-rich motifs in peptides that relate to anti-microbial activity are conserved (149) and as artificial tags are indeed exploited in subcellular targeting (150). Non-removal of histidine tags after expression of recombinant proteins for the purpose of testing anti-microbial activity bears inherent problems, and findings have to be viewed with utmost caution (151–154). Awareness of this potential pitfall was raised in a very pertinent article in 2013 (155).

By contrast, proteolytic cleavage of high molecular weight kininogen during bacterial infection generates an internal peptide, which has antibacterial activity that compares to LL-37 (156). Similarly, in bovine plasma, activated kallikrein releases from high molecular weight kininogen a histidine-rich fragment (157). Nordahl et al. (152) demonstrated effective antibacterial activity of a histidine-rich peptide generated from high molecular weight kininogen. However, the effect may be potentiated by the presence of the uncleaved histidine tag.

## Conclusion

In conclusion, much is to be learnt from cross-specialty comparisons.

Apart from refining one’s experimental design (cave histidine tags), greater clarity was gained in the use of the term “anti-microbial peptide.” Often, an analog (functionally similar gene product), not homolog (shared ancestry) is meant, and sometimes, a recombinantly expressed or proteolytically generated section only of a protein.

While having important functions in maintaining tissue homeostasis, anti-microbial peptides and complement are both involved in shaping the immune response and transcend from the purely innate immunity realm to adjuvant the adaptive immune response.

**Figure 1:**
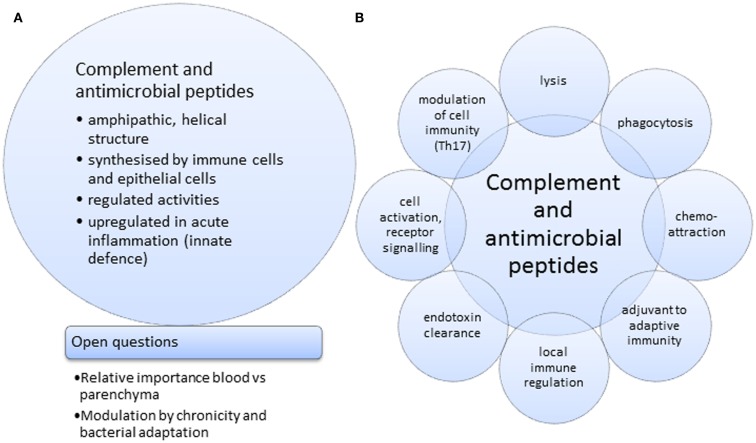
**Joint features (A) and activities (B) for complement and anti-microbial peptides**.

**Figure 2:**
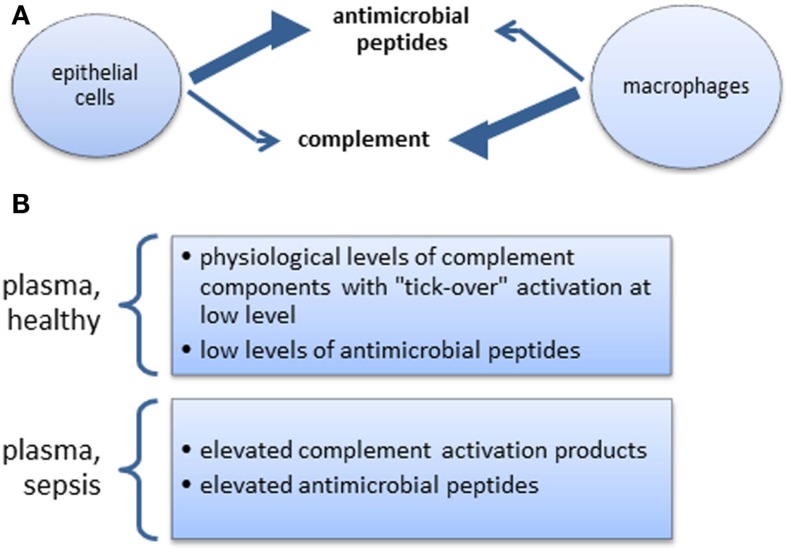
**(A)** Steady state skewed tissue resident cell-type-specific production of anti-microbial peptides and complement. The extent of relative changes of these productions during inflammation is not well documented. **(B)** Complement and anti-microbial peptides in systemic circulation.

In many aspects of health and disease, complement and anti-microbial peptides are remarkably similar in function, sharing certain features and broad range of activities (Figures 1A,B). They may, however, operate at differing preponderance in separate niches, e.g., blood/tissue, epithelial cells/macrophages (Figures 2A,B), supporting the view that two specialist systems are operating in a complementary way. In the context of beneficial activity of immune modulators applied clinically in sepsis, such as Vitamin D (158) and more recently omega-3 fatty acid preparations (159), parallel measurements of, e.g., C3 and LL-37, produced by cells, which express Vitamin D receptor (VDR) and ω-3 fatty acid receptor (GPR120), would provide the type of comparative analyses needed to direct this overlapping field.

## Conflict of Interest Statement

The authors declare that the research was conducted in the absence of any commercial or financial relationships that could be construed as a potential conflict of interest.

## References

[1] Rus H Cudrici C Niculescu F. The role of the complement system in innate immunity. Immunol Res (2005) 33:103–12. 10.1385/IR:33:2:103 16234578

[2] Hajishengallis G Lambris JD. Crosstalk pathways between toll-like receptors and the complement system. Trends Immunol (2010) 31:154–63. 10.1016/j.it.2010.01.002 20153254 PMC2849859

[3] Zipfel PF Skerka C. Complement regulators and inhibitory proteins. Nat Rev Immunol (2009) 9:729–40. 10.1038/nri2620 19730437

[4] Thurman JM Renner B. Dynamic control of the complement system by modulated expression of regulatory proteins. Lab Invest (2011) 91:4–11. 10.1038/labinvest.2010.173 20921948 PMC3109904

[5] Kouser L Abdul-Aziz M Nayak A Stover CM Sim RB Kishore U. Properdin and factor h: opposing players on the alternative complement pathway “see-saw”. Front Immunol (2013) 4:93. 10.3389/fimmu.2013.00093 23630525 PMC3632793

[6] Harwig SS Park AS Lehrer RI. Characterization of defensin precursors in mature human neutrophils. Blood (1992) 79:1532–7. 1547345

[7] Wilde CG Snable JL Griffith JE Scott RW. Characterization of two azurophil granule proteases with active-site homology to neutrophil elastase. J Biol Chem (1990) 265:2038–41. 2404977

[8] Sengeløv H Follin P Kjeldsen L Lollike K Dahlgren C Borregaard N. Mobilization of granules and secretory vesicles during in vivo exudation of human neutrophils. J Immunol (1995) 154:4157–65. 7535822

[9] Jones DE Bevins CL. Paneth cells of the human small intestine express an antimicrobial peptide gene. J Biol Chem (1992) 267:23216–25. 1429669

[10] O’Neil DA Porter EM Elewaut D Anderson GM Eckmann L Ganz T Expression and regulation of the human beta-defensins hBD-1 and hBD-2 in intestinal epithelium. J Immunol (1999) 163:6718–24. 10586069

[11] Menzies BE Kenoyer A. *Staphylococcus aureus* infection of epidermal keratinocytes promotes expression of innate antimicrobial peptides. Infect Immun (2005) 73:5241–4. 10.1128/IAI.73.8.5241-5244.2005 16041048 PMC1201242

[12] Wehkamp J Stange EF. Paneth cells and the innate immune response. Curr Opin Gastroenterol (2006) 22:644–50. 10.1097/01.mog.0000245541.95408.86 17053443

[13] Zanger P Holzer J Schleucher R Steffen H Schittek B Gabrysch S. Constitutive expression of the antimicrobial peptide RNase 7 is associated with *Staphylococcus aureus* infection of the skin. J Infect Dis (2009) 200:1907–15. 10.1086/648408 19919305

[14] Röhrl J Yang D Oppenheim JJ Hehlgans T. Specific binding and chemotactic activity of mBD4 and its functional orthologue hBD2 to CCR6-expressing cells. J Biol Chem (2010) 285:7028–34. 10.1074/jbc.M109.091090 20068036 PMC2844152

[15] De Paula VS Pomin VH Valente AP. Unique properties of human β-defensin 6 (hBD6) and glycosaminoglycan complex: sandwich-like dimerization and competition with the chemokine receptor 2 (CCR2) binding site. J Biol Chem (2014) 289:22969–79. 10.1074/jbc.M114.572529 24970887 PMC4132797

[16] De Yang Chen Q Schmidt AP Anderson GM Wang JM Wooters J LL-37, the neutrophil granule- and epithelial cell-derived cathelicidin, utilizes formyl peptide receptor-like 1 (FPRL1) as a receptor to chemoattract human peripheral blood neutrophils, monocytes, and T cells. J Exp Med (2000) 192:1069–74. 10.1084/jem.192.7.1069 11015447 PMC2193321

[17] Elssner A Duncan M Gavrilin M Wewers MD. A novel P2X7 receptor activator, the human cathelicidin-derived peptide LL37, induces IL-1 beta processing and release. J Immunol (2004) 172:4987–94. 10.4049/jimmunol.172.8.4987 15067080

[18] Lau YE Rozek A Scott MG Goosney DL Davidson DJ Hancock REW. Interaction and cellular localization of the human host defense peptide LL-37 with lung epithelial cells. Infect Immun (2005) 73:583–91. 10.1128/IAI.73.1.583-591.2005 15618198 PMC538997

[19] Morris KM Aden DP Knowles BB Colten HR. Complement biosynthesis by the human hepatoma-derived cell line HepG2. J Clin Invest (1982) 70:906–13. 10.1172/JCI110687 6288774 PMC370299

[20] Morgan BP Gasque P Extrahepatic complement biosynthesis: where, when and why? Clin Exp Immunol (1997) 107:1–7 10.1046/j.1365-2249.1997.d01-890.x 9010248 PMC1904545

[21] Mandal MNA Ayyagari R. Complement factor H: spatial and temporal expression and localization in the eye. Invest Ophthalmol Vis Sci (2006) 47:4091–7. 10.1167/iovs.05-1655 16936129

[22] Johnson E Hetland G Mononuclear phagocytes have the potential to synthesize the complete functional complement system. Scand J Immunol (1988) 27:489–93 10.1111/j.1365-3083.1988.tb02375.x 3287594

[23] Garred P Hetland G Mollnes TE Stoervold G. Synthesis of C3, C5, C6, C7, C8, and C9 by human fibroblasts. Scand J Immunol (1990) 32:555–60. 10.1111/j.1365-3083.1990.tb03196.x 2270435

[24] Langeggen H Pausa M Johnson E Casarsa C Tedesco F. The endothelium is an extrahepatic site of synthesis of the seventh component of the complement system. Clin Exp Immunol (2000) 121:69–76. 10.1046/j.1365-2249.2000.01238.x 10886241 PMC1905676

[25] Brooimans RA Stegmann AP van Dorp WT van der Ark AA van der Woude FJ van Es LA Interleukin 2 mediates stimulation of complement C3 biosynthesis in human proximal tubular epithelial cells. J Clin Invest (1991) 88:379–84. 10.1172/JCI115314 1864952 PMC295341

[26] Andoh A Fujiyama Y Sakumoto H Uchihara H Kimura T Koyama S Detection of complement C3 and factor B gene expression in normal colorectal mucosa, adenomas and carcinomas. Clin Exp Immunol (1998) 111:477–83. 10.1046/j.1365-2249.1998.00496.x 9528886 PMC1904873

[27] Griffiths MR Neal JW Fontaine M Das T Gasque P. Complement factor H, a marker of self protects against experimental autoimmune encephalomyelitis. J Immunol (2009) 182:4368–77. 10.4049/jimmunol.0800205 19299737

[28] Pattrick M Luckett J Yue L Stover C. Dual role of complement in adipose tissue. Mol Immunol (2009) 46:755–60. 10.1016/j.molimm.2008.09.013 18954909

[29] Hosszu KK Valentino A Ji Y Matkovic M Pednekar L Rehage N Cell surface expression and function of the macromolecular c1 complex on the surface of human monocytes. Front Immunol (2012) 3:38. 10.3389/fimmu.2012.00038 22566921 PMC3342062

[30] Camous L Roumenina L Bigot S Brachemi S Frémeaux-Bacchi V Lesavre P Complement alternative pathway acts as a positive feedback amplification of neutrophil activation. Blood (2011) 117:1340–9. 10.1182/blood-2010-05-283564 21063021

[31] Whaley K. Biosynthesis of the complement components and the regulatory proteins of the alternative complement pathway by human peripheral blood monocytes. J Exp Med (1980) 151:501–16. 10.1084/jem.151.3.501 6444659 PMC2185797

[32] Bongrazio M Pries AR Zakrzewicz A. The endothelium as physiological source of properdin: role of wall shear stress. Mol Immunol (2003) 39:669–75. 10.1016/S0161-5890(02)00215-8 12493642

[33] Kemper C Mitchell LM Zhang L Hourcade DE. The complement protein properdin binds apoptotic T cells and promotes complement activation and phagocytosis. Proc Natl Acad Sci USA (2008) 105:9023–8. 10.1073/pnas.0801015105 18579773 PMC2449358

[34] Xu W Berger SP Trouw LA de Boer HC Schlagwein N Mutsaers C Properdin binds to late apoptotic and necrotic cells independently of C3b and regulates alternative pathway complement activation. J Immunol (2008) 180:7613–21. 10.4049/jimmunol.180.11.7613 18490764

[35] Kemper C Atkinson JP Hourcade DE. Properdin: emerging roles of a pattern-recognition molecule. Annu Rev Immunol (2010) 28:131–55. 10.1146/annurev-immunol-030409-101250 19947883

[36] Grailer JJ Bosmann M Ward PA. Regulatory effects of C5a on IL-17A, IL-17F, and IL-23. Front Immunol (2012) 3:387. 10.3389/fimmu.2012.00387 23316190 PMC3540403

[37] Lajoie S Lewkowich IP Suzuki Y Clark JR Sproles AA Dienger K Complement-mediated regulation of the IL-17A axis is a central genetic determinant of the severity of experimental allergic asthma. Nat Immunol (2010) 11:928–35. 10.1038/ni.1926 20802484 PMC2943538

[38] Tang S Zhou W Sheerin NS Vaughan RW Sacks SH. Contribution of renal secreted complement C3 to the circulating pool in humans. J Immunol (1999) 162:4336–41. 10201966

[39] Laufer J Katz Y Passwell JH. Extrahepatic synthesis of complement proteins in inflammation. Mol Immunol (2001) 38:221–9. 10.1016/S0161-5890(01)00044-X 11532283

[40] Kolev M Friec GL Kemper C. Complement – tapping into new sites and effector systems. Nat Rev Immunol (2014) 14:811–20. 10.1038/nri3761 25394942

[41] Ouellette AJ Hsieh MM Nosek MT Cano-Gauci DF Huttner KM Buick RN Mouse Paneth cell defensins: primary structures and antibacterial activities of numerous cryptdin isoforms. Infect Immun (1994) 62:5040–7. 7927786 10.1128/iai.62.11.5040-5047.1994 PMC303224

[42] Geyer G Lysozyme in Paneth cell secretions. Acta Histochem (1973) 45:126–32. 4197859

[43] Mulherkar R Rao RS Wagle AS Patki V Deo MG Enhancing factor, a Paneth cell specific protein from mouse small intestines: predicted amino acid sequence from RT-PCR amplified cDNA and its expression. Biochem Biophys Res Commun (1993) 197:351–2 10.1006/bbrc.1993.2485 8250944

[44] Selsted ME Miller SI Henschen AH Ouellette AJ. Enteric defensins: antibiotic peptide components of intestinal host defense. J Cell Biol (1992) 118:929–36. 10.1083/jcb.118.4.929 1500431 PMC2289569

[45] Ouellette AJ. Paneth cell alpha-defensin synthesis and function. Curr Top Microbiol Immunol (2006) 306:1–25. 10.1007/3-540-29916-5_1 16909916

[46] Ouellette AJ. Paneth cells and innate immunity in the crypt microenvironment. Gastroenterology (1997) 113:1779–84. 10.1053/gast.1997.v113.pm9352884 9352884

[47] Saberwal G Nagaraj R. Cell-lytic and antibacterial peptides that act by perturbing the barrier function of membranes: facets of their conformational features, structure-function correlations and membrane-perturbing abilities. Biochim Biophys Acta (1994) 1197:109–31. 10.1016/0304-4157(94)90002-7 8031824

[48] Hancock JT. Superoxide, hydrogen peroxide and nitric oxide as signalling molecules: their production and role in disease. Br J Biomed Sci (1997) 54:38–46. 9167306

[49] Müller-Eberhard HJ The membrane attack complex of complement. Annu Rev Immunol (1986) 4:503–28 10.1146/annurev.immunol.4.1.503 3518749

[50] Lovelace LL Cooper CL Sodetz JM Lebioda L. Structure of human C8 protein provides mechanistic insight into membrane pore formation by complement. J Biol Chem (2011) 286:17585–92. 10.1074/jbc.M111.219766 21454577 PMC3093833

[51] Pasupuleti M Schmidtchen A Malmsten M. Antimicrobial peptides: key components of the innate immune system. Crit Rev Biotechnol (2012) 32:143–71. 10.3109/07388551.2011.594423 22074402

[52] Laarman A Milder F van Strijp J Rooijakkers S. Complement inhibition by gram-positive pathogens: molecular mechanisms and therapeutic implications. J Mol Med (2010) 88:115–20. 10.1007/s00109-009-0572-y 20062962 PMC2832872

[53] Nizet V. Antimicrobial peptide resistance mechanisms of human bacterial pathogens. Curr Issues Mol Biol (2006) 8:11–26. 16450883

[54] Pasupuleti M Walse B Nordahl EA Mörgelin M Malmsten M Schmidtchen A. Preservation of antimicrobial properties of complement peptide C3a, from invertebrates to humans. J Biol Chem (2007) 282:2520–8. 10.1074/jbc.M607848200 17132627

[55] Sonesson A Ringstad L Nordahl EA Malmsten M Mörgelin M Schmidtchen A. Antifungal activity of C3a and C3a-derived peptides against Candida. Biochim Biophys Acta (2007) 1768:346–53. 10.1016/j.bbamem.2006.10.017 17169328

[56] Grupp A Kimmel M Fritz P Voggenreiter B Stöltzing H Kuhlmann U The expression patterns of peritoneal defensins. Perit Dial Int (2007) 27:654–62. 17984427

[57] Bazargani F Albrektsson A Yahyapour N Braide M. Low molecular weight heparin improves peritoneal ultrafiltration and blocks complement and coagulation. Perit Dial Int (2005) 25:394–404. 16022098

[58] Zarrinkalam KH Leavesley DI Stanley JM Atkins GJ Faull RJ. Expression of defensin antimicrobial peptides in the peritoneal cavity of patients on peritoneal dialysis. Perit Dial Int (2001) 21:501–8. 11757835

[59] Wan M van der Does AM Tang X Lindbom L Agerberth B Haeggström JZ. Antimicrobial peptide LL-37 promotes bacterial phagocytosis by human macrophages. J Leukoc Biol (2014) 95:971–81. 10.1189/jlb.0513304 24550523

[60] Honczarenko M Ratajczak MZ Nicholson-Weller A Silberstein LE. Complement C3a enhances CXCL12 (SDF-1)-mediated chemotaxis of bone marrow hematopoietic cells independently of C3a receptor. J Immunol (2005) 175:3698–706. 10.4049/jimmunol.175.6.3698 16148115

[61] Dobó J Major B Kékesi KA Szabó I Megyeri M Hajela K Cleavage of kininogen and subsequent bradykinin release by the complement component: mannose-binding lectin-associated serine protease (MASP)-1. PLoS One (2011) 6:e20036. 10.1371/journal.pone.0020036 21625439 PMC3100311

[62] Kohidai L Kovács K Csaba G. Direct chemotactic effect of bradykinin and related peptides-significance of amino- and carboxyterminal character of oligopeptides in chemotaxis of tetrahymena pyriformis. Cell Biol Int (2002) 26:55–62. 10.1006/cbir.2001.0809 11779221

[63] Oppenheim JJ Biragyn A Kwak LW Yang D. Roles of antimicrobial peptides such as defensins in innate and adaptive immunity. Ann Rheum Dis (2003) 62(Suppl 2):ii17–21. 10.1136/ard.62.suppl_2.ii17 14532141 PMC1766745

[64] Dempsey PW Allison ME Akkaraju S Goodnow CC Fearon DT. C3d of complement as a molecular adjuvant: bridging innate and acquired immunity. Science (1996) 271:348–50. 10.1126/science.271.5247.348 8553069

[65] Glovsky MM Ward PA Johnson KJ Complement determinations in human disease. Ann Allergy Asthma Immunol (2004) 93:513–22 10.1016/S1081-1206(10)61257-4 15609759

[66] Hancock RE Diamond G. The role of cationic antimicrobial peptides in innate host defences. Trends Microbiol (2000) 8:402–10. 10.1016/S0966-842X(00)01823-0 10989307

[67] Koyasu S Moro K Role of innate lymphocytes in infection and inflammation. Front Immunol (2012) 3:101 10.3389/fimmu.2012.00101 22783250 PMC3346161

[68] Liang SC Tan X-Y Luxenberg DP Karim R Dunussi-Joannopoulos K Collins M Interleukin (IL)-22 and IL-17 are coexpressed by Th17 cells and cooperatively enhance expression of antimicrobial peptides. J Exp Med (2006) 203:2271–9. 10.1084/jem.20061308 16982811 PMC2118116

[69] Weaver DJ Reis ES Pandey MK Köhl G Harris N Gerard C C5a receptor-deficient dendritic cells promote induction of Treg and Th17 cells. Eur J Immunol (2010) 40:710–21. 10.1002/eji.200939333 20017191 PMC3040298

[70] Asgari E Le Friec G Yamamoto H Perucha E Sacks SS Köhl J C3a modulates IL-1β secretion in human monocytes by regulating ATP efflux and subsequent NLRP3 inflammasome activation. Blood (2013) 122:3473–81. 10.1182/blood-2013-05-502229 23878142

[71] Dupont A Mohamed F Salehen N Glenn S Francescut L Adib R Septicaemia models using *Streptococcus pneumoniae* and *Listeria monocytogenes*: understanding the role of complement properdin. Med Microbiol Immunol (2014) 203:257–71. 10.1007/s00430-013-0324-z 24728387 PMC4118039

[72] Fang C Zhang X Miwa T Song W-C. Complement promotes the development of inflammatory T-helper 17 cells through synergistic interaction with toll-like receptor signaling and interleukin-6 production. Blood (2009) 114:1005–15. 10.1182/blood-2009-01-198283 19491392 PMC2721782

[73] van der Does AM Joosten SA Vroomans E Bogaards SJP van Meijgaarden KE Ottenhoff THM The antimicrobial peptide hLF1-11 drives monocyte-dendritic cell differentiation toward dendritic cells that promote antifungal responses and enhance Th17 polarization. J Innate Immun (2012) 4:284–92. 10.1159/000332941 22261275 PMC6741610

[74] Cunningham PN Holers VM Alexander JJ Guthridge JM Carroll MC Quigg RJ Complement is activated in kidney by endotoxin but does not cause the ensuing acute renal failure. Kidney Int (2000) 58:1580–7 10.1046/j.1523-1755.2000.00319.x 11012892

[75] Song WC Crosstalk between complement and toll-like receptors. Toxicol Pathol (2012) 40:174–82 10.1177/0192623311428478 22109714

[76] Giuliani A Pirri G Rinaldi AC. Antimicrobial peptides: the LPS connection. Methods Mol Biol (2010) 618:137–54. 10.1007/978-1-60761-594-1_10 20094863

[77] Ren J Zhao Y Yuan Y Han G Li W Huang Q Complement depletion deteriorates clinical outcomes of severe abdominal sepsis: a conspirator of infection and coagulopathy in crime? PLoS One (2012) 7:e47095. 10.1371/journal.pone.0047095 23091606 PMC3473032

[78] Book M Chen Q Lehmann LE Klaschik S Weber S Schewe JC Inducibility of the endogenous antibiotic peptide beta-defensin 2 is impaired in patients with severe sepsis. Crit Care (2007) 11:R19. 10.1186/cc5694 17302973 PMC2151902

[79] Amrein K Zajic P Schnedl C Waltensdorfer A Fruhwald S Holl A Vitamin D status and its association with season, hospital and sepsis mortality in critical illness. Crit Care (2014) 18:R47. 10.1186/cc13790 24661739 PMC4057427

[80] Wang TT Nestel FP Bourdeau V Nagai Y Wang Q Liao J Cutting edge: 1,25-dihydroxyvitamin D3 is a direct inducer of antimicrobial peptide gene expression. J Immunol (2004) 173:2909–12. 10.4049/jimmunol.173.10.6490-c 15322146

[81] Littman BH Sanders KM. Effects of vitamin D3 and IFN-gamma on the synthesis of the second complement component, C2, by a human myeloid leukemia (HL-60) cell line. J Immunol (1988) 140:3082–5. 2834451

[82] Tsukamoto H Nagasawa K Ueda Y Mayumi T Furugo I Tsuru T Effects of cell differentiation on the synthesis of the third and fourth component of complement (C3, C4) by the human monocytic cell line U937. Immunology (1992) 77:621–3. 1337336 PMC1421655

[83] Riedemann NC Guo R-F Neff TA Laudes IJ Keller KA Sarma VJ Increased C5a receptor expression in sepsis. J Clin Invest (2002) 110:101–8 10.1172/JCI200215409 12093893 PMC151030

[84] Daigneault M Preston JA Marriott HM Whyte MKB Dockrell DH. The identification of markers of macrophage differentiation in PMA-stimulated THP-1 cells and monocyte-derived macrophages. PLoS One (2010) 5:e8668. 10.1371/journal.pone.0008668 20084270 PMC2800192

[85] Bengio S Gilbert D Peulve P Daveau M Fontaine M. Biosynthesis of the third component of complement (C3) by the human monocytic-cell line U-937. Induction by phorbol myristate acetate. Biochem J (1986) 239:711–6. 3827822 10.1042/bj2390711 PMC1147344

[86] Strunk RC Kunke KS Giclas PC. Human peripheral blood monocyte-derived macrophages produce haemolytically active C3 in vitro. Immunology (1983) 49:169–74. 6840804 PMC1454087

[87] Høgåsen AK Abrahamsen TG Heparin suppresses lipopolysaccharide-induced monocyte production of several cytokines, but simultaneously stimulates C3 production. Thromb Res (1995) 80:179–84 10.1016/0049-3848(95)00164-M 8588195

[88] Cole FS Schneeberger EE Lichtenberg NA Colten HR. Complement biosynthesis in human breast-milk macrophages and blood monocytes. Immunology (1982) 46:429–41. 6919505 PMC1555385

[89] Minta JO Isenman DE. Biosynthesis of the third component of complement by the human monocyte-like cell line, U-937. Mol Immunol (1987) 24:1105–11. 10.1016/0161-5890(87)90079-4 3316990

[90] Goodrum KJ. Complement component C3 secretion by mouse macrophage-like cell lines. J Leukoc Biol (1987) 41:295–301. 3471827 10.1002/jlb.41.4.295

[91] Mogilenko DA Kudriavtsev IV Trulioff AS Shavva VS Dizhe EB Missyul BV Modified low density lipoprotein stimulates complement C3 expression and secretion via liver X receptor and Toll-like receptor 4 activation in human macrophages. J Biol Chem (2012) 287:5954–68. 10.1074/jbc.M111.289322 22194611 PMC3285363

[92] van Furth R van Schadewijk-Nieuwstad M Elzenga-Claasen I Cornelisse C Nibbering P. Morphological, cytochemical, functional, and proliferative characteristics of four murine macrophage-like cell lines. Cell Immunol (1985) 90:339–57. 10.1016/0008-8749(85)90199-6 3967303

[93] Tavano R Segat D Gobbo M Papini E. The honeybee antimicrobial peptide apidaecin differentially immunomodulates human macrophages, monocytes and dendritic cells. J Innate Immun (2011) 3:614–22. 10.1159/000327839 21677421

[94] Van der Does AM Bogaards SJP Ravensbergen B Beekhuizen H van Dissel JT Nibbering PH. Antimicrobial peptide hLF1-11 directs granulocyte-macrophage colony-stimulating factor-driven monocyte differentiation toward macrophages with enhanced recognition and clearance of pathogens. Antimicrob Agents Chemother (2010) 54:811–6. 10.1128/AAC.00652-09 19933796 PMC2812139

[95] Van der Does AM Beekhuizen H Ravensbergen B Vos T Ottenhoff THM van Dissel JT LL-37 directs macrophage differentiation toward macrophages with a proinflammatory signature. J Immunol (2010) 185:1442–9. 10.4049/jimmunol.1000376 20610648

[96] Fairweather D Cihakova D. Alternatively activated macrophages in infection and autoimmunity. J Autoimmun (2009) 33:222–30. 10.1016/j.jaut.2009.09.012 19819674 PMC2783278

[97] Fischer MB Ma M Hsu NC Carroll MC. Local synthesis of C3 within the splenic lymphoid compartment can reconstitute the impaired immune response in C3-deficient mice. J Immunol (1998) 160:2619–25. 9510159

[98] Celada A Klemsz MJ Maki RA. Interferon-gamma activates multiple pathways to regulate the expression of the genes for major histocompatibility class II I-A beta, tumor necrosis factor and complement component C3 in mouse macrophages. Eur J Immunol (1989) 19:1103–9. 10.1002/eji.1830190621 2502420

[99] Mitchell TJ Naughton M Norsworthy P Davies KA Walport MJ Morley BJ. IFN-gamma up-regulates expression of the complement components C3 and C4 by stabilization of mRNA. J Immunol (1996) 156:4429–34. 8666817

[100] Böttger EC Metzger S Bitter-Suermann D Stevenson G Kleindienst S Burger R. Impaired humoral immune response in complement C3-deficient guinea pigs: absence of secondary antibody response. Eur J Immunol (1986) 16:1231–5. 10.1002/eji.1830161008 2945728

[101] Brown KL Poon GFT Birkenhead D Pena OM Falsafi R Dahlgren C Host defense peptide LL-37 selectively reduces proinflammatory macrophage responses. J Immunol (2011) 186:5497–505. 10.4049/jimmunol.1002508 21441450

[102] Mookherjee N Brown KL Bowdish DME Doria S Falsafi R Hokamp K Modulation of the TLR-mediated inflammatory response by the endogenous human host defense peptide LL-37. J Immunol (2006) 176:2455–64. 10.4049/jimmunol.176.4.2455 16456005

[103] Bruns H Fabri M Maurberger A Pasemann S Fahrenwaldt C Wilke A M1 macrophages eliminate lymphoma cells through the vitamin D dependent antimicrobial peptide cathelicidin. American Society of Hematology 53^rd^ Annual meeting Dec 10-13. San Diego, USA (2011).

[104] Ginhoux F Jung S. Monocytes and macrophages: developmental pathways and tissue homeostasis. Nat Rev Immunol (2014) 14:392–404. 10.1038/nri3671 24854589

[105] Skattum L van Deuren M van der Poll T Truedsson L. Complement deficiency states and associated infections. Mol Immunol (2011) 48:1643–55. 10.1016/j.molimm.2011.05.001 21624663

[106] Ricklin D Lambris JD Complement in immune and inflammatory disorders: pathophysiological mechanisms. J Immunol (2013) 190:3831–8 10.4049/jimmunol.1203200 23564577 PMC3623009

[107] Lachmann PJ Smith RAG. Taking complement to the clinic – has the time finally come? Scand J Immunol (2009) 69:471–8. 10.1111/j.1365-3083.2009.02258.x 19439007

[108] Tzou P Reichhart J-M Lemaitre B. Constitutive expression of a single antimicrobial peptide can restore wild-type resistance to infection in immunodeficient *Drosophila* mutants. Proc Natl Acad Sci USA (2002) 99:2152–7. 10.1073/pnas.042411999 11854512 PMC122334

[109] Iimura M Gallo RL Hase K Miyamoto Y Eckmann L Kagnoff MF. Cathelicidin mediates innate intestinal defense against colonization with epithelial adherent bacterial pathogens. J Immunol (2005) 174:4901–7. 10.4049/jimmunol.174.8.4901 15814717

[110] Huang LC Reins RY Gallo RL McDermott AM. Cathelicidin-deficient (*Cnlp*^-/-^) mice show increased susceptibility to *Pseudomonas aeruginosa* keratitis. Invest Ophthalmol Vis Sci (2007) 48:4498–508. 10.1167/iovs.07-0274 17898271 PMC4234056

[111] Chromek M Arvidsson I Karpman D. The antimicrobial peptide cathelicidin protects mice from *Escherichia coli* O157:H7-mediated disease. PLoS One (2012) 7:e46476. 10.1371/journal.pone.0046476 23077510 PMC3471911

[112] Kovach MA Ballinger MN Newstead MW Zeng X Bhan U Yu F Cathelicidin-related antimicrobial peptide is required for effective lung mucosal immunity in Gram-negative bacterial pneumonia. J Immunol (2012) 189:304–11. 10.4049/jimmunol.1103196 22634613 PMC3566644

[113] Merres J Höss J Albrecht L-J Kress E Soehnlein O Jansen S Role of the cathelicidin-related antimicrobial peptide in inflammation and mortality in a mouse model of bacterial meningitis. J Innate Immun (2014) 6:205–18. 10.1159/000353645 23969854 PMC6741491

[114] Pütsep K Carlsson G Boman HG Andersson M. Deficiency of antibacterial peptides in patients with Morbus Kostmann: an observation study. Lancet (2002) 360:1144–9. 10.1016/S0140-6736(02)11201-3 12387964

[115] Rieg S Steffen H Seeber S Humeny A Kalbacher H Dietz K Deficiency of dermcidin-derived antimicrobial peptides in sweat of patients with atopic dermatitis correlates with an impaired innate defense of human skin *in vivo*. J Immunol (2005) 174:8003–10. 10.4049/jimmunol.174.12.8003 15944307

[116] de Haar SF Hiemstra PS van Steenbergen MTJM Everts V Beertsen W. Role of polymorphonuclear leukocyte-derived serine proteinases in defense against *Actinobacillus actinomycetemcomitans*. Infect Immun (2006) 74:5284–91. 10.1128/IAI.02016-05 16926422 PMC1594863

[117] Ehrnthaller C Ignatius A Gebhard F Huber-Lang M. New insights of an old defense system: structure, function, and clinical relevance of the complement system. Mol Med (2011) 17:317–29. 10.2119/molmed.2010.00149 21046060 PMC3060978

[118] Rittirsch D Redl H Huber-Lang M. Role of complement in multiorgan failure. Clin Dev Immunol (2012) 2012:962927. 10.1155/2012/962927 23320021 PMC3539671

[119] Charchaflieh J Wei J Labaze G Hou YJ Babarsh B Stutz H The role of complement system in septic shock. Clin Dev Immunol (2012) 2012:1–8 10.1155/2012/407324 PMC3459296 23049598

[120] Hack CE Nuijens JH Felt-Bersma RJ Schreuder WO Eerenberg-Belmer AJ Paardekooper J Elevated plasma levels of the anaphylatoxins C3a and C4a are associated with a fatal outcome in sepsis. Am J Med (1989) 86:20–6. 10.1016/0002-9343(89)90224-6 2783358

[121] Gerard C Complement C5a in the sepsis syndrome – too much of a good thing? N Engl J Med (2003) 348:167–9 10.1056/NEJMcibr022995 12519929

[122] Hammerschmidt DE Weaver LJ Hudson LD Craddock PR Jacob HS. Association of complement activation and elevated plasma-C5a with adult respiratory distress syndrome. Pathophysiological relevance and possible prognostic value. Lancet (1980) 1:947–9. 10.1016/S0140-6736(80)91403-8 6103300

[123] Collins HL Bancroft GJ. Cytokine enhancement of complement-dependent phagocytosis by macrophages: synergy of tumor necrosis factor-alpha and granulocyte-macrophage colony-stimulating factor for phagocytosis of *Cryptococcus neoformans*. Eur J Immunol (1992) 22:1447–54. 10.1002/eji.1830220617 1601035

[124] Oikonomopoulou K Ricklin D Ward PA Lambris JD Interactions between coagulation and complement – their role in inflammation. Semin Immunopathol (2012) 34:151–65 10.1007/s00281-011-0280-x 21811895 PMC3372068

[125] Pedersen ED Waje-Andreassen U Vedeler CA Aamodt G Mollnes TE. Systemic complement activation following human acute ischaemic stroke. Clin Exp Immunol (2004) 137:117–22. 10.1111/j.1365-2249.2004.02489.x 15196251 PMC1809093

[126] van Beek J Elward K Gasque P. Activation of complement in the central nervous system: roles in neurodegeneration and neuroprotection. Ann N Y Acad Sci (2003) 992:56–71. 10.1111/j.1749-6632.2003.tb03138.x 12794047

[127] Osaka H McGinty A Höepken UE Lu B Gerard C Pasinetti GM. Expression of C5a receptor in mouse brain: role in signal transduction and neurodegeneration. Neuroscience (1999) 88:1073–82. 10.1016/S0306-4522(98)00372-8 10336122

[128] Stahel PF Morganti-Kossmann MC Kossmann T The role of the complement system in traumatic brain injury. Brain Res Brain Res Rev (1998) 27:243–56 10.1016/S0165-0173(98)00015-0 9729408

[129] Lynch NJ Willis CL Nolan CC Roscher S Fowler MJ Weihe E Microglial activation and increased synthesis of complement component C1q precedes blood-brain barrier dysfunction in rats. Mol Immunol (2004) 40:709–16. 10.1016/j.molimm.2003.08.009 14644096

[130] Arumugam TV Shiels IA Woodruff TM Granger DN Taylor SM The role of the complement system in ischemia-reperfusion injury. Shock (2004) 21:401–9 10.1097/00024382-200405000-00002 15087815

[131] Link C Hawlisch H Meyer zu Vilsendorf A Gylerüz S Nagel E Köhl J. Selection of phage-displayed anti-guinea pig C5 or C5a antibodies and their application in xenotransplantation. Mol Immunol (1999) 36:1235–47. 10.1016/S0161-5890(99)00135-2 10684963

[132] Stahl GL Xu Y Hao L Miller M Buras JA Fung M Role for the alternative complement pathway in ischemia/reperfusion injury. Am J Pathol (2003) 162:449–55. 10.1016/S0002-9440(10)63839-4 12547703 PMC1851150

[133] Langlois PF Gawryl MS. Accentuated formation of the terminal C5b-9 complement complex in patient plasma precedes development of the adult respiratory distress syndrome. Am Rev Respir Dis (1988) 138:368–75. 10.1164/ajrccm/138.2.368 3264125

[134] Mathey D Schofer J Schäfer HJ Hamdoch T Joachim HC Ritgen A Early accumulation of the terminal complement-complex in the ischaemic myocardium after reperfusion. Eur Heart J (1994) 15:418–23. 8013522 10.1093/oxfordjournals.eurheartj.a060516

[135] Väkevä A Morgan BP Tikkanen I Helin K Laurila P Meri S. Time course of complement activation and inhibitor expression after ischemic injury of rat myocardium. Am J Pathol (1994) 144:1357–68. 7515561 PMC1887457

[136] Buono C Come CE Witztum JL Maguire GF Connelly PW Carroll M Influence of C3 deficiency on atherosclerosis. Circulation (2002) 105:3025–31 10.1161/01.CIR.0000019584.04929.83 12081998

[137] Francescut L Steiner T Byrne S Cianflone K Francis S Stover C. The role of complement in the development and manifestation of murine atherogenic inflammation: novel avenues. J Innate Immun (2012) 4:260–72. 10.1159/000332435 22116497 PMC6741550

[138] Hertle E van Greevenbroek MM Arts IC van der Kallen CJ Geijselaers SL Feskens EJ Distinct associations of complement C3a and its precursor C3 with atherosclerosis and cardiovascular disease. The CODAM study. Thromb Haemost (2014) 111:1102–11. 10.1160/TH13-10-0831 24500020

[139] Liu F Wu L Wu G Wang C Zhang L Tomlinson S Targeted mouse complement inhibitor CR2-Crry protects against the development of atherosclerosis in mice. Atherosclerosis (2014) 234:237–43. 10.1016/j.atherosclerosis.2014.03.004 24685815 PMC4267679

[140] Davidson DJ Currie AJ Reid GSD Bowdish DME MacDonald KL Ma RC The cationic antimicrobial peptide LL-37 modulates dendritic cell differentiation and dendritic cell-induced T cell polarization. J Immunol (2004) 172:1146–56. 10.4049/jimmunol.172.2.1146 14707090

[141] Stockinger S Albers T Duerr CU Ménard S Pütsep K Andersson M Interleukin-13-mediated Paneth cell degranulation and antimicrobial peptide release. J Innate Immun (2014) 6:530–41. 10.1159/000357644 24556597 PMC6741497

[142] Nizet V Ohtake T Lauth X Trowbridge J Rudisill J Dorschner RA Innate antimicrobial peptide protects the skin from invasive bacterial infection. Nature (2001) 414:454–7 10.1038/35106587 11719807

[143] Sørensen OE Cowland JB Theilgaard-Mönch K Liu L Ganz T Borregaard N. Wound healing and expression of antimicrobial peptides/polypeptides in human keratinocytes, a consequence of common growth factors. J Immunol (2003) 170:5583–9. 10.4049/jimmunol.170.11.5583 12759437

[144] Salzman NH Underwood MA Bevins CL. Paneth cells, defensins, and the commensal microbiota: a hypothesis on intimate interplay at the intestinal mucosa. Semin Immunol (2007) 19:70–83. 10.1016/j.smim.2007.04.002 17485224

[145] Arlt A Bharti R Ilves I Häsler R Miettinen P Paajanen H Characteristic changes in microbial community composition and expression of innate immune genes in acute appendicitis. Innate Immun (2015) 21:30–41. 10.1177/1753425913515033 24336024

[146] Nordahl EA Rydengård V Nyberg P Nitsche DP Mörgelin M Malmsten M Activation of the complement system generates antibacterial peptides. Proc Natl Acad Sci U S A (2004) 101:16879–84. 10.1073/pnas.0406678101 15550543 PMC534732

[147] Malmsten M Schmidtchen A Antimicrobial C3a – biology, biophysics, and evolution. Adv Exp Med Biol (2007) 598:141–58 10.1007/978-0-387-71767-8_11 17892210

[148] Rydengård V Andersson Nordahl E Schmidtchen A. Zinc potentiates the antibacterial effects of histidine-rich peptides against *Enterococcus faecalis*. FEBS J (2006) 273:2399–406. 10.1111/j.1742-4658.2006.05246.x 16704414

[149] Lai R Takeuchi H Lomas LO Jonczy J Rigden DJ Rees HH A new type of antimicrobial protein with multiple histidines from the hard tick, *Amblyomma hebraeum*. FASEB J (2004) 18:1447–9. 10.1096/fj.03-1154fje 15247144

[150] Ferrer-Miralles N Corchero JL Kumar P Cedano JA Gupta KC Villaverde A Biological activities of histidine-rich peptides; merging biotechnology and nanomedicine. Microb Cell Fact (2011) 10:101. 10.1186/1475-2859-10-101 22136342 PMC3339332

[151] Singh PK Tack BF McCray PB Welsh MJ. Synergistic and additive killing by antimicrobial factors found in human airway surface liquid. Am J Physiol Lung Cell Mol Physiol (2000) 279:L799–805. 11053013 10.1152/ajplung.2000.279.5.L799

[152] Nordahl EA Rydengård V Mörgelin M Schmidtchen A. Domain 5 of high molecular weight kininogen is antibacterial. J Biol Chem (2005) 280:34832–9. 10.1074/jbc.M507249200 16091369

[153] Qu H Chen B Peng H Wang K. Molecular cloning, recombinant expression, and antimicrobial activity of EC-hepcidin3, a new four-cysteine hepcidin isoform from *Epinephelus coioides*. Biosci Biotechnol Biochem (2013) 77:103–10. 10.1271/bbb.120600 23291752

[154] Ali YM Hayat A Saeed BM Haleem KS Alshamrani S Kenawy HI Low-dose recombinant properdin provides substantial protection against *Streptococcus pneumoniae* and *Neisseria meningitidis* infection. Proc Natl Acad Sci USA (2014) 111:5301–6. 10.1073/pnas.1401011111 24706855 PMC3986141

[155] Rowinska-Zyrek M Witkowska D Potocki S Remelli M Kozlowski H His-rich sequences – is plagiarism from nature a good idea? New J Chem (2013) 37:58 10.1039/c2nj40558j

[156] Frick I-M Akesson P Herwald H Mörgelin M Malmsten M Nägler DK The contact system – a novel branch of innate immunity generating antibacterial peptides. EMBO J (2006) 25:5569–78. 10.1038/sj.emboj.7601422 17093496 PMC1679765

[157] Han YN Komiya M Iwanaga S Suzuki T. Studies on the primary structure of bovine high-molecular-weight kininogen. Amino acid sequence of a fragment (“histidine-rich peptide”) released by plasma kallikrein. J Biochem (1975) 77:55–68. 1169237

[158] Jeng L Yamshchikov AV Judd SE Blumberg HM Martin GS Ziegler TR Alterations in vitamin D status and anti-microbial peptide levels in patients in the intensive care unit with sepsis. J Transl Med (2009) 7:28. 10.1186/1479-5876-7-28 19389235 PMC2684740

[159] Bilku D Zimmer J Hall T Chung W Stover C Dennison A Effect of parenteral omega-3 fish oil on C3 levels and mortality in septic patients on intensive care unit. Int J Surg (2013) 11:655 10.1016/j.ijsu.2013.06.368

